# Alu RNA Structural Features Modulate Immune Cell Activation and A-to-I Editing of Alu RNAs Is Diminished in Human Inflammatory Bowel Disease

**DOI:** 10.3389/fimmu.2022.818023

**Published:** 2022-01-20

**Authors:** Thomas M. Aune, John T. Tossberg, Rachel M. Heinrich, Krislyn P. Porter, Philip S. Crooke

**Affiliations:** ^1^ Department of Medicine, Vanderbilt University Medical Center, Nashville, TN, United States; ^2^ Department of Pathology, Microbiology and Immunology, Vanderbilt University Medical Center, Nashville, TN, United States; ^3^ Department of Mathematics, Vanderbilt University, Nashville, TN, United States

**Keywords:** Alu RNA, A-to-I editing, IRF responses, NF-kB responses, SINE, autoimmune disease, viral disease

## Abstract

Alu retrotransposons belong to the class of short interspersed nuclear elements (SINEs). Alu RNA is abundant in cells and its repetitive structure forms double-stranded RNAs (dsRNA) that activate dsRNA sensors and trigger innate immune responses with significant pathological consequences. Mechanisms to prevent innate immune activation include deamination of adenosines to inosines in dsRNAs, referred to as A-to-I editing, degradation of Alu RNAs by endoribonucleases, and sequestration of Alu RNAs by RNA binding proteins. We have previously demonstrated that widespread loss of Alu RNA A-to-I editing is associated with diverse human diseases including viral (COVID-19, influenza) and autoimmune diseases (multiple sclerosis). Here we demonstrate loss of A-to-I editing in leukocytes is also associated with inflammatory bowel diseases. Our structure-function analysis demonstrates that ability to activate innate immune responses resides in the left arm of Alu RNA, requires a 5’-PPP, RIG-I is the major Alu dsRNA sensor, and A-to-I editing disrupts both structure and function. Further, edited Alu RNAs inhibit activity of unedited Alu RNAs. Altering Alu RNA nucleotide sequence increases biological activity. Two classes of Alu RNAs exist, one class stimulates both IRF and NF-kB transcriptional activity and a second class only stimulates IRF transcriptional activity. Thus, Alu RNAs play important roles in human disease but may also have therapeutic potential.

## Introduction

Alu elements belong to the class of short interspersed nuclear elements (SINEs), are unique to primates, and arose from a head-to-tail fusion of 7SL RNA (1–3). Over one million copies of Alu retrotransposons are dispersed throughout the human genome, they are about 300 bp in length and therefore make up about 10% of the human genome. Alu genomic elements are divided into classes named after investigators who discovered the individual classes; AluJ represents the oldest class in evolutionary time dating back 65 million years; the AluS class dates back to about 30 million years, and AluY represents the youngest class, which has the greatest ability to undergo transposition (4, 5). Most of the older classes have sufficiently mutated so they have little ability to undergo transposition. The basic structure of an Alu element consists of a left arm and right arm of similar nucleotide sequence representing the two original 7SL RNA sequences joined by an A-rich linker. Alu elements also contain two short promoter boxes both located in the left arm termed the 5’ A box and the 3’ B box that are involved in Alu replication and mobilization (6–8). Rodents have a similar class of SINEs referred to as B1 elements that arose from a single 7SL RNA thus consisting only of the left arm (9). Rodents contain additional SINE elements, referred to as B2, B3, and B4 classes, that arose from transfer RNAs.

The majority of Alu elements in human genomes are located in introns and 3’ and 5’ untranslated regions (UTR) of protein-coding genes. Thus, Alu elements are transcribed by RNA pol2 as part of a pre-mRNA but are also transcribed by RNA pol3 as part of their normal life cycle. Alu RNAs are also abundant in cells. Because of their repetitive nucleotide sequence, Alu RNAs have the capacity to form double-stranded RNAs (dsRNA) that can stimulate dsRNA sensors, including TLR3, RIG-I, MDA5, resulting in activation of IRF and NF-kB transcriptional paths and induction of IFNs, interferon-stimulated genes (ISGs), NF-kB regulated genes, pro-inflammatory cytokines and other mediators creating something akin to the host ‘anti-viral’ response (10–19).

Eukaryotic cells possess multiple mechanisms to prevent this unwanted activation of dsRNA sensors by endogenous Alu dsRNA thereby preventing potentially dangerous downstream effects (13, 20–22). One example is deamination of adenosines to inosines in dsRNA, termed A-to-I editing and catalyzed by the adenosine deaminase, ADAR (23–29). In general terms, A-to-I editing of Alu dsRNAs is thought to disrupt dsRNAs structure preventing their recognition by dsRNA sensors. Deletion of ADAR in mice causes a dramatic increase in expression of IFNs, ISGs, other pro-inflammatory mediators and is embryonic lethal. Inactivating ADAR mutations in humans create a similar pro-inflammatory response and cause one form of Aicardi-Goutières syndrome which, in humans, may result in severe encephalopathy and can lead to either existence in a vegetative state or even death (30). In addition, various RNA-binding proteins both positively and negatively contribute to overall A-to-I editing levels and their dysregulation may also have pathogenic consequences. A second example is the endoribonuclease, Dicer. The canonical role of Dicer is to process pre-microRNAs to mature microRNAs. Dicer also degrades Alu dsRNAs and loss of Dicer in retinal epithelium causes accumulation of Alu dsRNAs and cell death and is associated with development of one form of macular degeneration (31, 32). A third example is TDP-43, an RNA binding protein that participates in multiple steps of RNA metabolism; dysregulation of TDP-43 is associated with multiple neurological disorders, including frontotemporal lobar dementia and amyotrophic lateral sclerosis. In cell models, loss of TDP-43 results in accumulation of dsRNAs, including Alu dsRNAs, and stimulation of a RIG-I dependent immune response and cell death (33).

**Figure 1:**
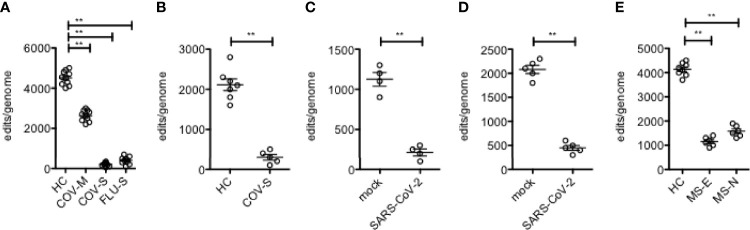
Loss of A-to-I editing of Alu RNAs in response to viral infection. **(A)** Edits/genome in leukocytes (Y-axis) isolated from healthy controls and patients with mild or severe COVID 19 disease or with severe influenza (X-axis). **(B)** As in **(A)**, except RNA for analysis was isolated from lung biopsies. **(C)** As in **(A)**, except purified human DC were mock infected or infected with SARS-CoV-2. **(D)** As in **(A)**, except NHBE were mock infected or infected with SARS-CoV-2. **(E)** As in **(A)**, except leukocytes were isolated from HC or patients with MS, either established disease (MS-E) or at the time of diagnosis who were treatment naïve (MS-N). **P < 0.001, unpaired t-test with Welch’s correction.

We have previously demonstrated reduced levels of A-to-I editing in leukocytes obtained from people with relapsing remitting multiple sclerosis [MS] and concomitant elevation of levels of Alu dsRNAs and ISGs (12, 34). We have also found that mild and severe COVID-19 diseases are also associated with moderate and substantial loss of A-to-I editing of Alu RNAs, respectively (10, 35). Similarly, severe influenza disease (requiring hospitalization) is also associated with substantial loss of A-to-I editing of Alu RNAs (10). Infection of dendritic epithelial cells with SARS-Cov-2 in tissue culture also induces rapid loss of Alu RNAs A-to-I editing (summarized in **Figures 1A–E**).

Like MS, causes of persistent activation of the immune response in inflammatory bowel diseases are not well understood (36). In experimental models, loss of ADAR induces endoplasmic reticulum stress and activation of IFN responses disrupting cellular homeostasis (24, 28). For these reasons, we asked if A-to-I editing of endogenous RNAs may be disrupted in human inflammatory bowel disease. In the current study, we demonstrate that two inflammatory bowel diseases, ulcerative colitis (UC) and Crohn’s disease (Cr), also result in leukocyte loss of A-to-I editing. In contrast, Celiac disease (Ce), believed to result from damage to the small intestine by an immune response to ingested gluten (37), and irritable bowel disease (IBS), the cause of which is not well understood (38), are not associated with substantial loss or gain of Alu dsRNA A-to-I editing. We propose that decreased A-to-I editing of Alu dsRNAs contributes to elevated inflammatory responses observed in these diverse diseases. Since Alu dsRNAs are such potent agonists of innate immune responses, we performed additional studies to better delineate relationships between Alu RNA functional activities and underlying nucleotide sequences or structure.

## Methods

### Study Populations and Sample Collection

Studies involving patients with COVID and severe influenza infection included HC (healthy controls, N=7), COV-M (mild COVID-19 disease, N=7), COV-S (severe COVID-19 disease, N=7) and Flu-S (severe influenza infection requiring hospitalization, N=7) cohorts (GSE149689) and a separate study with HC (N=7) and COV-S (N=5) cohorts (GSE147507). Studies involving patients with multiple sclerosis [MS] included HC (N=8), MS-E (patients with established disease on various medications, N=8), and MS-N (patients at the time of initial diagnosis who were treatment naïve, N=8) (GSE126427). Studies involving patients with inflammatory bowel diseases and related syndromes included HC (N=8), ulcerative colitis (UC, N=6), Crohn’s (Cr, N=6), Celiac disease (Ce, N=6) and irritable bowel syndrome (IBS, N=6) (GSE126427). Leukocytes and lung biopsies were tissue sources for the GSE149689 and GSE147507 studies, respectively. Leukocytes were also tissue sources for MS and IBD studies. Isolated human dendritic cells (N=4, GSE144106) or normal human lung epithelial cells (NHBE) (N=6, GSE147507) were infected with SARS-CoV-2 in tissue culture. RNA was harvested after 24 hr. and processed for RNA-sequencing (39–41).

### A-to-I Editing

We obtained whole genome RNA-sequencing FASTQ files from the Gene Expression Omnibus and employed the following workflow to identify endogenous RNA A-to-I–editing sites from paired FASTQ sequencing files essentially as previously described. The main identification tool was a python-based package called the SPRINT toolkit (42) that accepts sequence files and produces text files with the following information for each edit site: 1) genomic location; 2) type of edit (e.g., A-to-G; T-to-C), strand (+ or -); 3) number of edits per site and total number of reads per editing site. Mathematica programs were developed to synthesize data: numbers of samples in groups with unique and shared editing sites, mean numbers of total reads and edits for each editing site, editing sites common and unique to group pairs, and editing sites per gene. This information was tied to an Alu database to annotate each site: gene locations (intronic, noncoding RNA, intergenic, 5’ and 3’ UTRs) and if editing sites resided in Alu or non-Alu elements (43). To create genome-wide A-to-I–editing indices, we identified all A-to-I–editing sites present in one sample and summed edit/read ratios for all editing sites across the genome for each sample within a case or control cohort. To guard against sequencing errors, we required that editing sites had ≥5 total reads per site and an edit/read ratio ≥0.05. Gene expression levels from RNA-seq FASTQ files were determined using the DESeq R package as described (44). Both raw p-values and p-values after adjusting for multiple testing, false discovery rate (FDR), were determined.

### Synthesis and Testing of Alu RNAs

Alu DNA sequences were from the GrCh37 (hg19) assembly. We designed unedited Alu DNA templates and changed A nucleotides edited in HC but not disease cohorts to G nucleotides as mimics of A-to-I editing. A SP6 promoter was added to the 5’-end and synthetic double stranded DNA templates were obtained from IDT (34, 45). RNA transcription was performed using Megascript SP6 (InvivoGen) essentially as previously described. THP-1 reporter cell lines (InvivoGen) contained stably integrated luciferase genes under the control of either an IFN-stimulated response element (ISRE) or NF-kB response element were employed to measure cellular responses to Alu RNAs. HEK293 reporter cells contained a stably integrated luciferase gene under the control of ISRE with or without a stably integrated DDX58 gene that encodes the dsRNA sensor, RIG-I (wildtype HEK293 cells express the dsRNA sensor TLR3 but not dsRNA sensors RIG-I and MDA5). Transfections were performed using Lipofectamine RNAiMAX (Thermo Fisher Scientific) (12). Luciferase activity was determined after 24 h using luciferin substrate (InvivoGen) and light emission measured with a TD20/20 luminometer.

### Statistics

False discovery rates (FDR) were determined to correct for multiple testing using the DESeq R package. Unpaired t tests with Welch’s correction were used to determine statistical significance for nonparametric data. The Kruskal-Wallis test or one-way ANOVA was used to compare two or more independent samples of equal or different sizes. Dunn’s multiple comparison test was used to identify means significant from others. The two-way ANOVA was used to compare mean differences between groups segregated on two independent variables. χ^2^ analysis or Fisher’s exact test were used to determine if nonrandom associations existed between two categorical variables. These data were analyzed using GraphPad Prism.

## Results

### Reduced A-to-I Editing of Alu RNAs in Response to Viral or Autoimmune Disease

We have previously employed the SPRINT software package to analyze differences in levels of A-to-I editing of endogenous Alu RNAs in viral diseases; mild and severe COVID-19 disease in both leukocytes and lung biopsies and severe influenza (requiring hospitalization) in leukocytes (10, 35), as well as the autoimmune disease, relapsing remitting multiple sclerosis [MS] (34), both in patients before onset of therapies (MS-naïve, MS-N) and with established disease of >3 years duration (MS-established, MS-E). Salient results are summarized here (**Figure 1**). Briefly, COVID-19 disease leads to loss of Alu RNA A-to-I editing in both leukocytes and lung and degree of loss is proportional to disease severity. Severe influenza disease also resulted in loss of A-to-I editing similar in magnitude to that observed in severe COVID-19 disease. This is recapitulated in cell culture by infection with SARS-Cov-2. We measured differences in A-to-I editing of Alu RNAs in patients with MS and found loss of A-to-I editing in leukocytes. Thus, loss of A-to-I editing of Alu RNAs is associated with both viral and non-viral inflammatory disease.

### Altered A-to-I Editing in Inflammatory Bowel Diseases

**Figure 2:**
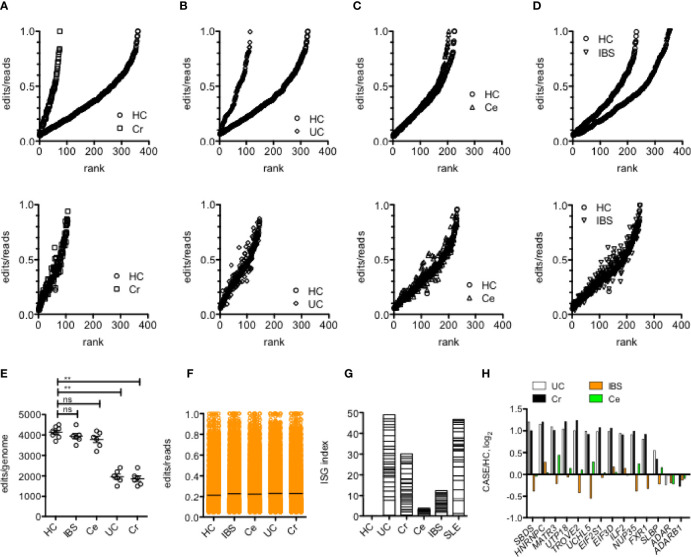
Reduced Alu RNA A-to-I editing in IBD. **(A)** Number of editing sites in leukocytes shared by all members of the HC cohort or all members of the Cr cohort (upper panel) or shared by all members of both HC and Cr cohorts (lower panel). Y-axis is average proportions of edits/reads for each editing site.X-axis is rank from lowest to highest proportion of edits/reads. P<0.001 χ^2^ analysis. **(B)** As in **(A)** except the comparison is between HC and UC. P<0.001 χ^2^ analysis. **(C)** As in **(A)**, except comparison is between HC and Ce. P=ns, χ^2^ analysis. **(D)** As in **(A)** except comparison is between HC and IBS. P=ns, χ^2^ analysis. **(E)** Average number of editing sites/genome in HC, IBS, Ce, UC and Cr. **P<0.001, unpaired t-test with Welch’s correction, ns=not significant. **(F)** Average proportion of edits/reads at all edited sites in HC, IBS, Ce, UC, and Cr. **(G)** An interferon-stimulated gene expression index was calculated by summing average expression ratios, case/HC, log_2_, of the following ISGs: *IFI16, IFI27, IFI27L1, IFI44, IFI44L, IFI6, IFIT1, IFIT5, IFITM1, IFITM3, OAS1, HLA-DRA, HLA-W, B2M, RPL10A, RPL11, RPL22, RPL23A, RPL32, RPL36, RPL36A, RPL36AL, RPL37, RPL39L, RPL4, RPL5, RPL6, RPL9*. **(H)** Genes encoding RNA-binding proteins that reduce A-to-I editing are over-expressed in UC and Cr. Expression levels of the indicated genes were determined from RNA-seq data. Y-axis is case/HC ratio, log_2_. Adjusted p<0.05 for each gene comparing UC or Cr to HC; adjusted p > 0.05 for each gene comparing IBS and Ce to HC.

We used several approaches to examine levels of A-to-I editing in leukocytes obtained from patients with inflammatory bowel diseases; Cr and UC, and from patients with Ce and IBS, compared to HC. To do so, we first identified the total number of common A-to-I editing sites defined as present in either all samples within the HC cohort or all samples in each disease cohort or that were present in all samples in both cohorts. We determined average proportion of edits/reads at each edited site. We found that the total number of common editing sites was reduced in Cr and UC compared to Ce, IBS and HC (**Figures 2A–D**, upper panels). Among common editing sites shared between case/control cohorts, proportions of edits/reads at each shared editing site were not different among case/control cohorts (**Figures 2A–D**, lower panels). We also calculated total edits/genome for each sample within each cohort and found that the total number of editing sites per genome was reduced in UC and Cr compared to HC, IBS and Ce (**Figure 2E**). The average proportions of edits/reads at all edited sites were not different among HC, IBS, Ce, UC, and Cr (**Figure 2F**). Our conclusion is that presence of UC and Cr resulted in a total loss of A-to-I editing at certain A-to-I editing sites rather than an overall reduction in proportion of edits/reads at editing sites.

In MS, loss of A-to-I editing results in accumulation of Alu dsRNAs and stimulation of the equivalent of an anti-viral response (12). Therefore, we compared expression levels of interferon-stimulated genes (ISGs) in the different disease cohorts. Overall, we found an increase in expression levels of ISGs in Cr and UC compared to Ce, IBS, and HC (**Figure 2G**). The ISG index in UC was similar in magnitude to the SLE ISG index. Thus, we conclude that common editing sites were lost in UC and Cr compared to Ce, IBS, and HC and that loss of editing is associated with increased expression of ISGs.

In addition to ADAR, levels of A-to-I editing are both positively and negatively regulated by an array of diverse RNA-binding proteins (RBPs) (46). We screened expression levels of genes that encode these various RBPs in HC, UC, Cr, IBS and Ce cohorts. We did not observe statistically significant differences in expression of genes that encode *ADAR* or *ADARB1* (**Figure 2H**). However, we identified 12 genes that were over-expressed in both UC and Cr cohorts compared to IBS, Ce and HC cohorts (adjusted P<0.05). Each of these genes encodes an RBP that, in at least one study, is a negative regulator of A-to-I editing (46). Thus, loss of A-to-I editing in UC and Cr may result from gain of expression of genes that encode RBPs that negatively impact A-to-I editing.

**Figure 3:**
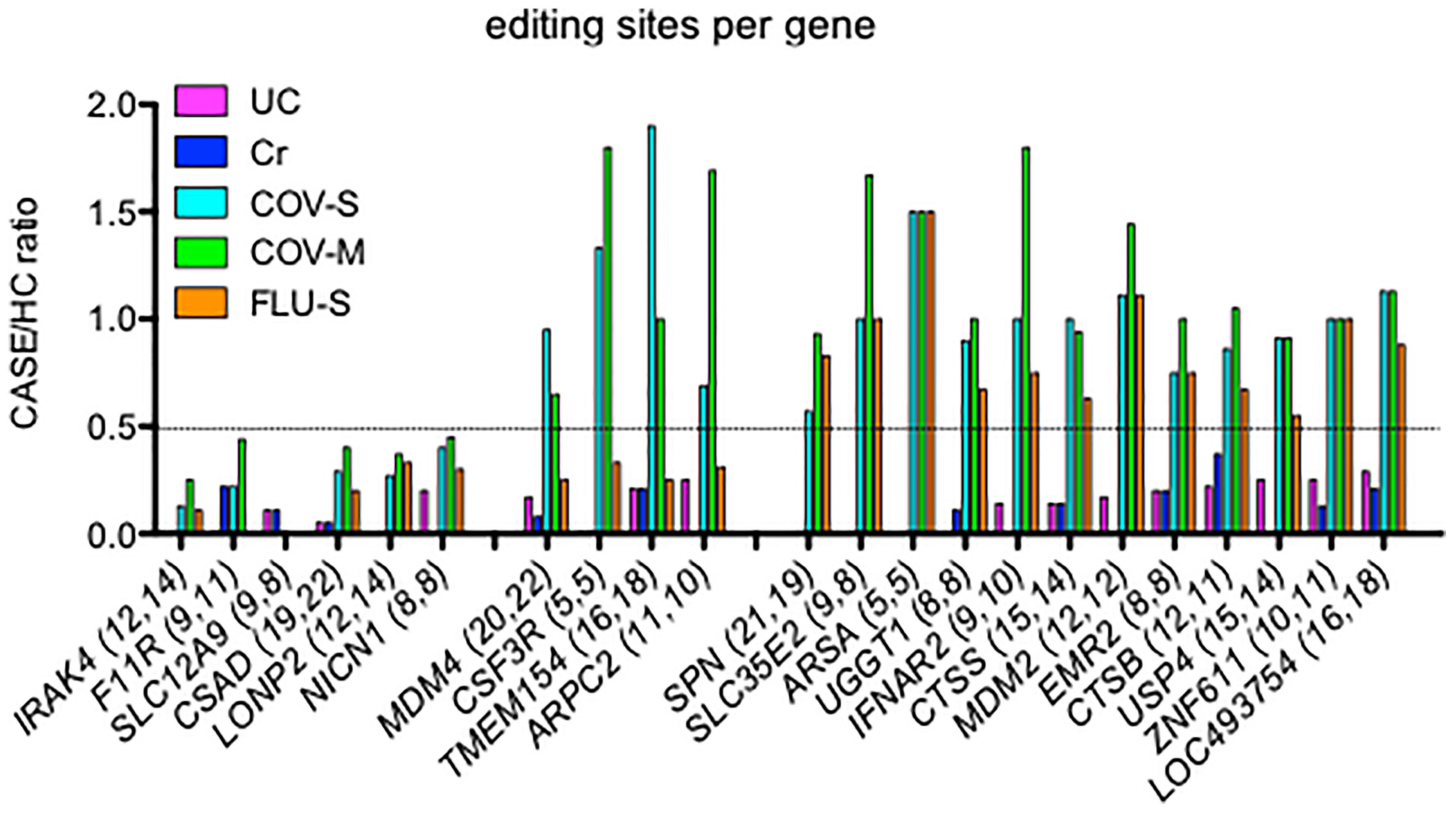
Comparison of lost editing sites among disease cohorts. We identified genes with the greatest loss of editing sites per gene in UC and Cr, expressed as CASE/HC ratio, Y-axis. We determined the degree of loss of editing sites per gene in COV-S, COV-M and FLU-S, also expressed as CASE/HC ratio. Number of editing sites per gene we detected in the two independent HC cohorts are shown in parentheses after each gene, X-axis.

Alu RNAs that undergo A-to-I editing are predominantly localized in intronic and 3’UTR gene regions (1, 43). In an attempt to compare loss of editing among UC, Cr, COV-S, COV-M and FLU-S cohorts, we identified genes with the greatest number of editing sites in the two independent HC cohorts and determined if loss of editing per gene was similar in the two classes of disease cohorts. Genes could be classified into those with reduced editing sites in all disease cohorts, *IRAK4*, *F11R*, *SLC12A9*, *CSAD*, *LONP2*, and *NICN1*, those with reduced editing sites in UC, Cr and FLU-S, but not COV-S and COV-M, *MDM4*, *CSF3R*, *TMEM154* and *ARPC2*, and those with reduced editing sites in UC and Cr but not COV-S, COV-M and FLU-S, *SPN*, *SLC35E2*, *ARSA*, *UGGT1*, *IFNAR2*, *CTSS*, *MDM2*, *EMR2*, *CTSB*, *USP4*, *ZNF611*, and *LOC493754* (**Figure 3**). Thus, we found shared loss of editing at certain genic sites among all disease cohorts while loss of editing at other genic sites was restricted to UC, Cr and FLU-S or just UC and Cr sample sets.

### Structure-Function Analysis of Alu RNAs

**Figure 4:**
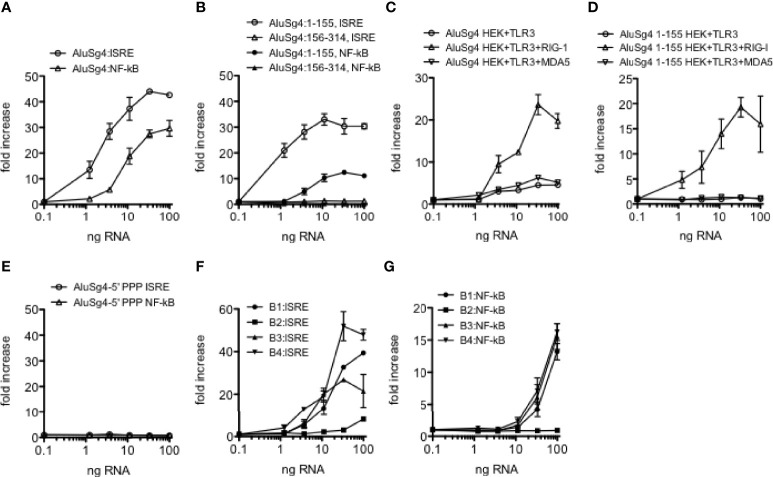
Structure-function analysis of Alu and B SINE RNAs. **(A)** Activation of IRF and NF-kB transcriptional responses in THP-1 cells by synthetic AluSg4 RNA. Y-axis is fold increase over mock-transfected controls. X-axis is ng/culture of AluSg4 RNA. ALuSg4 ISRE; P<0.0001 compared to mock-transfections, ALuSg4 NF-kB; P<0.0001 compared to mock-transfection. **(B)** As in **(A)** except activities of AluSg4 left arms (1-155) and right arms (154-314) were determined. P<0.0001: left arm, P=ns: right arm compared to mock-transfections. (**C, D**) Activation of dsRNA sensors by AluSg4 RNA. Complete length AluSg4 RNA or AluSg4 left arm RNA was transfected into HEK293 cells that naturally express TLR3 (HEK293+TLR3) or HEK293 with stably integrated DDX58 construct (HEK293+TLR3+RIG-I) or HEK293 transfected with an IFIH1 expression plasmid (HEK+TLR3+MDA5) also containing a stably integrated luciferase gene under the control of an ISRE. Y-axis is fold increase over mock-transfected controls. X-axis is ng/culture of AluSg4 RNA **(C)** or AluSg4 left arm RNA **(D)**. **(C)** P<0.001, two-way ANOVA compared to mock transfected controls for each transfection condition. **(D)** P < 0.001, two-way ANOVA, HEK+TLR3+RIG-I compared to mock-transfected controls, differences between other cell lines and mock-transfected controls were not significant. **(E)** 5’PPP is required for activation of IRF and NF-kB transcriptional responses by AluSg4. As in **(A)** except AluSg4 was treated with calf intestinal phosphatase to remove 5’PPP prior to transfection. P=ns compared to mock-transfections. **(F, G)** As in **(A)** except activation of IRF and NF-kB transcriptional responses by synthetic murine SINEs of the B1, B2, B3, and B4 classes were determined. P < 0.0001: B1, B2, or B3 compared to mock-transfections, P=ns: B4 compared to mock-transfections.

We synthesized intact Alu RNA elements containing both left and right arms, only the left or right arm, and monomeric B1, B2, B3 and B4 RNA elements (mouse) from double-stranded DNA templates and tested their ability to activate IRF and NF-kB transcriptional responses using THP-1 reporter cell lines containing a stably integrated luciferase gene under the control of either an interferon stimulated response element (ISRE) activated by IRF transcription factors or a NF-kB response element. We first tested an Alu RNA of the AluSg4 class located in the *MDM4* 3’ UTR that was edited in the HC cohort but unedited in both UC and Cr cohorts. We found that the intact Alu RNA was a potent stimulator of both IRF and NF-kB mediated transcriptional responses (**Figure 4A**). Second, we found that the Alu RNA left arm (AluSg4:1-155) stimulated IRF and NF-kB mediated transcriptional responses of similar magnitude to the intact Alu RNA but the Alu right arm RNA (AluSg4:156-314) lacked activity (**Figure 4B**). We analyzed five additional highly expressed Alu RNAs with strong stimulatory activity and found that activity resides only in the left arm (**Supplementary Figure 1**). Of these additional Alu RNAs tested, three belong to the AluJb class and two belong to the AluSx class. To identify dsRNA sensors activated by AluSg4 derived RNAs, we employed the HEK293 cell line containing a stably integrated luciferase gene under the control of an ISRE. HEK293 cells naturally express TLR3 but not DDX58 (encodes RIG-I) and IFIH1 (encodes MDA5) (HEK293+TLR3). We also analyzed a HEK293 cell line with a stably integrated DDX58 construct (HEK293+TLR3+RIG-I). To analyze activation of MDA5, we transfected an IFIH1 expression construct into HEK293 reporter cells. We found maximum activation of the IRF transcriptional response in the presence of both TLR3 and RIG-I compared to either TLR3 alone or TLR3 + MDA5 (**Figure 4C**). Complete AluSg4 RNA weakly activated the IRF transcriptional response in the presence of only TLR3 or TLR3+MDA5. We employed a similar strategy to analyze activation of dsRNA sensors by the AluSg4 left arm (AluSg4 1-155). We found substantial activation of the IRF transcriptional response in the HEK293+TLR3+RIG-I cell line but not in the other two cell lines under study (**Figure 4D**). We conclude that complete AluSg4 RNA activates IRF transcriptional responses *via* RIG-I and, to a lesser extent TLR3, while the shorter AluSG4 left arm RNA only activates IRF transcriptional responses *via* RIG-I.

Activation of RIG-I by viral ssRNAs or dsRNAs is reported to require a triphosphate group at the 5’ end of the RNA, 5’-PPP (47). Therefore, we asked if a 5’-PPP was also required for activity of AluSg4 RNAs by nuclease digestion of the synthesized Alu RNA. We found that the 5’-PPP was absolutely required for the intact Alu RNA to stimulate both IRF and NF-kB mediated transcriptional responses (**Figure 4E**). Thus, similar to what has been found for viral dsRNAs, activation of IRF and NF-kB transcriptional responses by AluSg4 RNA requires a 5’-PPP moiety.

We also tested ability of the mouse B1, B2, B3, and B4 RNAs we synthesized to stimulate IRF and NF-kB transcriptional responses. We found that members of the B1, B3, and B4 classes we selected stimulated transcriptional responses that were similar in magnitude to what we observed with the human AluSg4 RNA (**Figures 4F, G** **)**. The B2 RNA we selected lacked the ability to stimulate these transcriptional responses. Thus, both human Alu and mouse SINE RNAs are potent stimulators of IRF and NF-kB stimulated transcriptional activity, and activity of the human Alu RNA resides completely in the left arm.

### Activity of Unedited and Edited Alu RNAs

**Figure 5:**
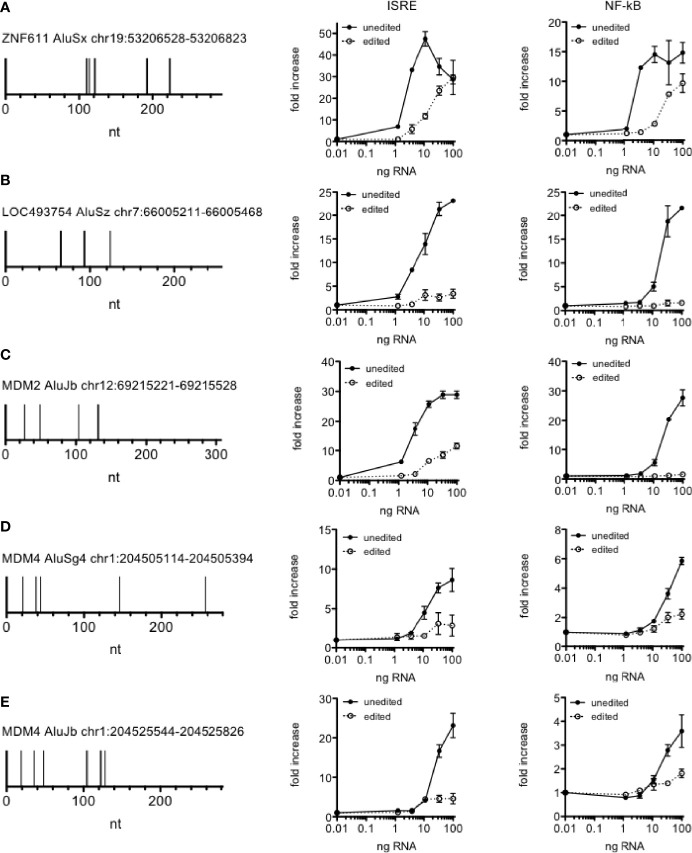
A-to-I editing of Alu RNAs abrogates activity. **(A-E)** Left panels show positions of A-to I edits (vertical spikes) in the indicated Alu RNAs observed in all samples in the HC cohort but sites were unedited in disease cohorts **(A)** COV-S, **(B)** Flu-S, **(C)** MS-N & MS-E, **(D)** UC, **(E)** Cr. Middle and left panels show activity of unedited and edited synthetic Alu RNAs in IRF and NF-kB reporter assays. P < 0.001 comparing each unedited and edited Alu RNAs **(A–E)**, two-way ANOVA.

An important function of A-to-I editing catalyzed by ADAR is to prevent recognition of Alu RNAs by dsRNA sensors, RIG-I, TLR3, and MDA5; loss of A-to-I editing of Alu RNAs results in accumulation of Alu dsRNAs, activation of dsRNA sensors and the triggering of downstream inflammatory responses as observed in both viral infection and autoimmune disease (24, 28). In the course of above studies, we identified Alu RNAs that were edited in HC, IBS and Ce cohorts but unedited in UC and Cr cohorts. We compared locations of edits in these different Alu elements. We also synthesized and tested activity of unedited and edited Alu RNAs in reporter assays. In general terms, we found that only about 5-8 adenosines were edited to inosines in each Alu RNA we examined of about 300 nt in total length (**Figures 5A–E**). We also found that these editing sites were not evenly distributed across the entire Alu RNA but were mostly clustered at the 3’ end of the left arm. This level of editing, only 5-8 edits per Alu RNA, was sufficient to reduce ability to stimulate both IRF and NF-kB transcriptional activity compared to the unedited Alu RNAs.

**Figure 6:**
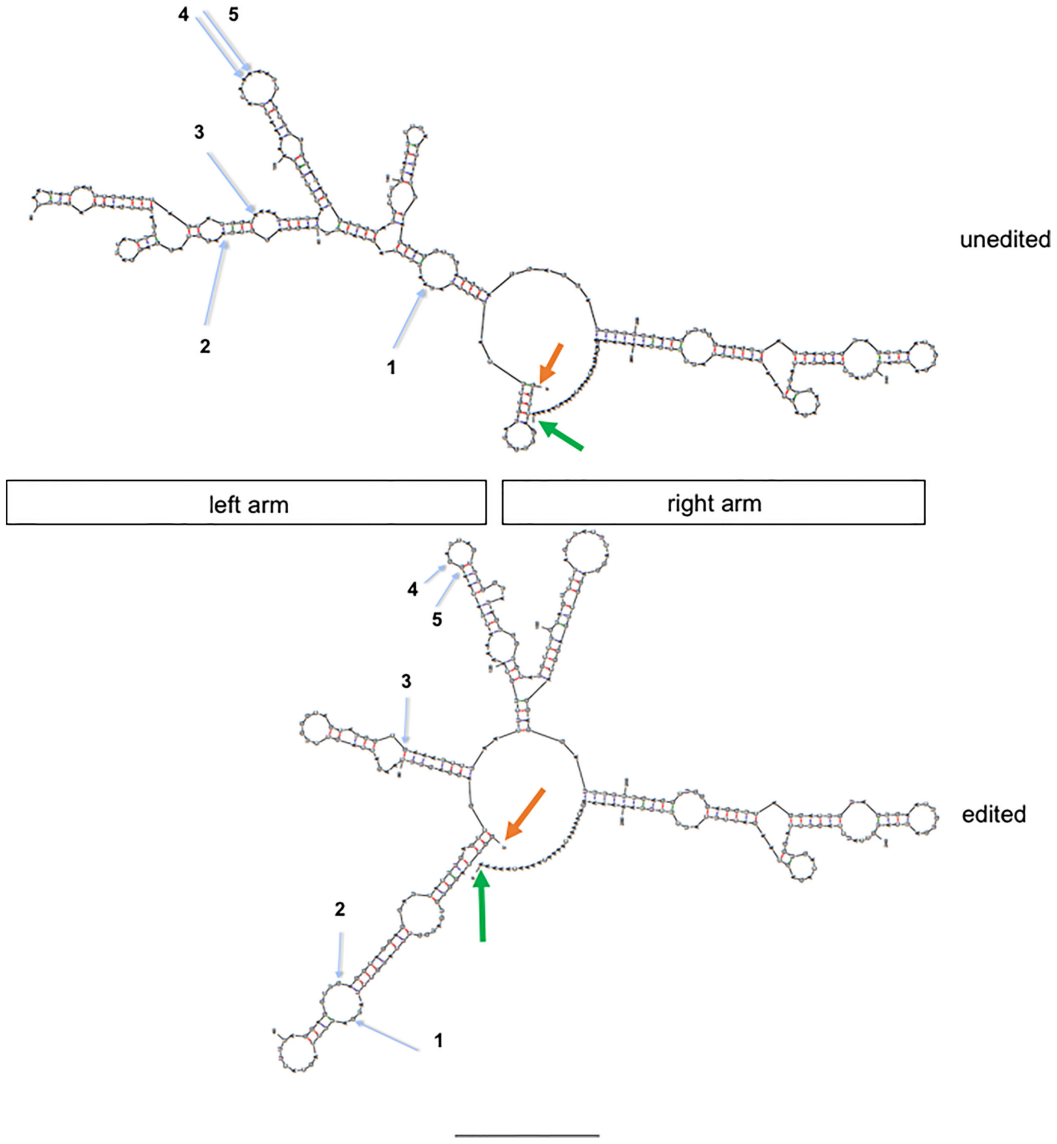
Structure prediction of unedited and edited MDM2 AluJb RNAs (genomic location: chr12:69215221-69215528. Orange arrows show 5’ end and green arrows show 3’ ends. Blue arrows show positions of A-to-I edits observed in the control but not case cohort numbered 1-5 from 5’ start to 3’ end. Unedited: nucleotide sequence from hg19; edited: indicated A’s (blue arrows) were replaced with G’s. Approximate locations of left and right arms are shown.

We compared predicted structures of one unedited and edited Alu RNA using the UNAfold web server (48). To create a mimic of the edited Alu RNA, we changed edited A’s to G’s and compared the predicted structures. We selected an Alu RNA with 5 A-to-I edits, positions identified by the blue arrows. We found that the unedited and edited Alu RNAs are predicted to have rather different secondary structures in the left arm and identical structures in the right arm (**Figure 6**). The major difference appeared to be that the unedited left arm possessed a more continuous dsRNA structure while the edited left arm appears to possess several shorter dsRNA structures. More in depth studies will be necessary to fully understand the structure-function relationships of edited and unedited Alu RNAs and how unedited and edited Alu dsRNAs are recognized by dsRNA sensors.

**Figure 7:**
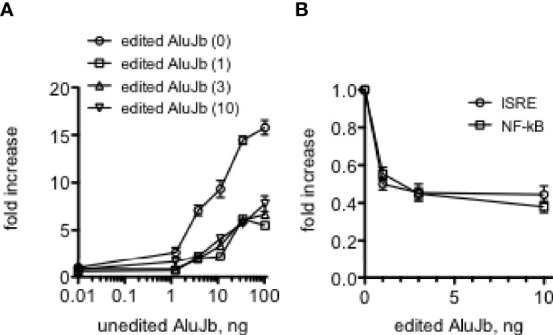
Edited AluJb RNA inhibit activation of transcriptional responses by unedited AluJb RNA. **(A)** THP-1 ISRE reporter cells were transfected with the indicated amounts of synthetic unedited AluJb RNA in the presence of 0, 1, 3, or 10 ng of synthetic edited AluJb RNA. Results are expressed as fold increase relative to mock-transfected controls. P<0.0001, two-way ANOVA comparing unedited AluJb RNA alone to either 1, 2, or 10 ng edited AluJb RNA, **(B)** Synthetic edited AluJb inhibits basal levels of ISRE and NF-kB activity. Indicated amounts of edited AluJb RNAs were transfected into THP-1 ISRE or NF-kB reporter cells. Results are expressed as fold increase relative to mock-transfected controls. P < 0.0001, both ISRE and NF-kB responses compared mock-transfected responses.

We also determined if the edited Alu RNA interfered with the ability of the almost identical unedited Alu RNA to activate IRF and NF-kB transcriptional responses. We found that small amounts of the relatively inactive edited Alu RNA inhibited the ability of unedited Alu RNA to fully activate IRF transcriptional responses (**Figure 7A**). We also found that small amounts of the edited Alu RNA actually lowered baseline IRF and NF-kB transcriptional activity (**Figure 7B**). Thus, both baseline and Alu RNA stimulated IRF and NF-kB transcriptional activity may be determined by the balance of unedited and edited Alu RNAs rather than just by the absolute levels of unedited Alu RNAs.

### Alu RNAs With Altered Biological Activity

**Figure 8:**
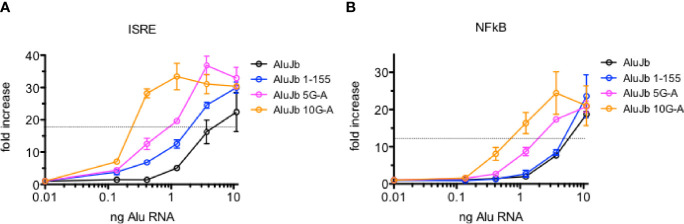
Alterations in AluJb nucleotide sequence increase activity. **(A, B)** Synthetic native AluJb, AluJb left arm (1-155) and AluJb left arm with 5 (5G-A) or 10 (10G-A) G’s changed to A’s were tested for their ability to activate ISRE **(A)** or NF-kB **(B)** transcriptional responses using THP-1 reporter cells. Results are expressed as fold increase relative to mock-transfected controls. P < 0.0001, two-way ANOVA both ISRA and NF-kB responses comparing AluJb 1-155 to AluJb 5G-A or AluJb 10G-A.

Given their biological properties, we have wondered if Alu RNAs may have therapeutic value, for example, as anti-viral agents, adjuvants for vaccines, or as adjuncts to cancer immunotherapy. Therefore, changing the activities of Alu RNAs may have therapeutic implications. Since changing A’s to G’s in the 3’ region of the Alu left arm resulted in loss of activity, we asked if changing G’s to A’s in this same region may result in gain of activity. We changed 5 or 10 G’s to A’s in the DNA template of an active Alu RNA and synthesized new novel Alu RNAs and tested their activity in the THP-1 reporter cell lines. We did not find that G to A changes resulted in an increase in the maximum level of activation of either IRF or NF-kB meditated transcriptional responses. Rather, we found that changing 10 G’s to A’s resulted in about a 10-fold decrease in EC_50_ or RNA amount required to achieve 50% maximal activation of either IRF or NF-kB transcriptional responses (**Figures 8A, B**). We observed somewhat less decreases in activity when only 5 G’s were changed to A’s. Taken together, these results suggest it may be possible to further engineer Alu RNAs to increase or decrease their biological activity.

**Figure 9:**
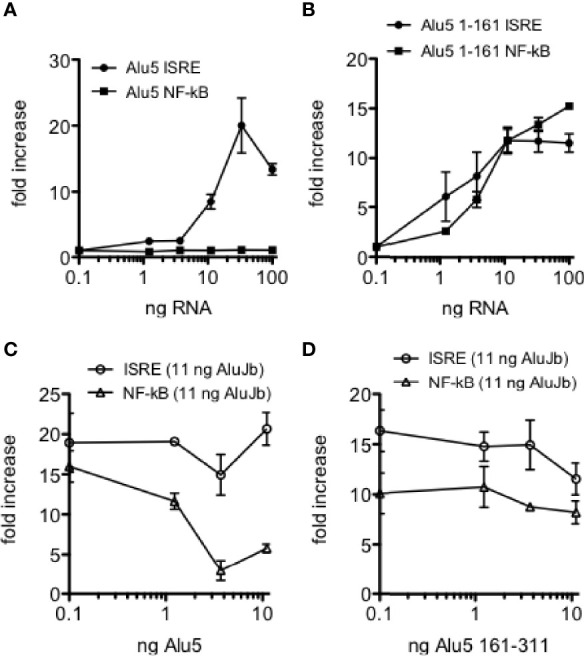
Selective activation of ISRE transcriptional responses by Alu5 RNA. **(A)** Indicated amounts of synthetic Alu5 RNA were transfected into THP-1 reporter cells. Results are expressed as fold increase relative to mock infected controls. P<0.0001, two-way ANOVA. **(B)** Alu5 left arm (1-161) activates both ISRE and NF-kB responses as in **(A)**. P=ns, two-way ANOVA. **(C)** Inhibition of AluJb-stimulated NF-kB, but not ISRE, transcriptional responses by Alu5 RNA. THP-1 reporter cells were transfected with 11 ng AluJb RNA and the indicated amounts of Alu5 RNA. Y-axis is fold increase relative to mock-transfected controls. P < 0.0001, two-way ANOVA. **(D)** As in **(C)**, but THP-1 reporter cells were transfected with 11 ng AluJb and the indicated amounts of the right arm of Alu5 (161-311).

We have identified a number of Alu RNAs that were edited in HC blood or lung biopsy samples but were unedited in disease cohorts; MS, COV-S, FLU-S, and IBD, synthesized unedited Alu RNAs from DNA templates, and tested their ability to activate IRF and NF-kB transcriptional responses. In general terms, unedited Alu RNAs activate both IRF and NF-kB transcriptional responses. However, we also identified a number of Alu RNAs that selectively activated IRF transcriptional responses and failed to activate NF-kB transcriptional responses. One example is shown, *CSFR*, AluJb class, chr1: 36,944,168-36,944,489, referred to as Alu5 (**Figure 9A**). However, when we synthesized and tested the Alu5 left arm, we found the Alu5 left arm activated both IRF and NF-kB transcriptional responses while the Alu5 right arm failed to activate IRF and NF-kB transcriptional responses (**Figure 9B**, and not shown). Overall, these results demonstrate that Alu RNAs also exist that activate IRF mediated transcriptional responses and not NF-kB transcriptional responses. The right arm activates both IRF and NF-kB transcriptional responses suggesting that the complete Alu RNA element may adopt a conformation that fails to activate or possibly inhibits NF-kB driven transcriptional responses and this inhibitory activity may require the intact Alu RNA or may exist in the right arm.

To distinguish among these two hypotheses, we transfected THP-1 reporter cell lines with the complete synthetic AluJb RNA that activates both IRF and NF-kB transcriptional responses either alone or in combination with varying amounts of either the complete Alu5 RNA or the Alu5 right arm (nt 162-311). For this experiment, we employed amounts of the complete AluJb RNA that induced maximal IRF and NF-kB driven transcriptional responses. We found that the complete Alu5 RNA had no significant effect on IRF driven transcriptional responses stimulated by AluJb RNA but markedly inhibited NF-kB driven transcriptional responses stimulated by AluJb RNA (**Figure 9C**). In contrast, the Alu5 right arm failed to significantly inhibit either IRF or NF-kB driven transcriptional responses stimulated by AluJb RNA (**Figure 9D**). Taken together, these results demonstrate that the complete Alu5 RNA is an effective inhibitor of NF-kB responses but not IRF responses stimulated by AluJb RNA. The Alu5 right arm alone is insufficient to inhibit NF-kB responses stimulated by AluJb.

## Discussion

In general terms, our studies bring together two major points. First, in several studies involving human participants, we find reduced levels of endogenous Alu RNA A-to-I editing in diverse human diseases including COV-S and FLU-S, as well as autoimmune diseases, MS, UC and Cr. Second, we find that unedited Alu RNAs, as found in these diseases, are potent activators of IRF and NF-kB transcriptional pathways but edited Alu RNAs, as found in HC, are only weakly active. Alu elements arose from a head to tail fusion of two 7SL RNA (8), often referred to as the left and right arms and we find that the stimulatory activity exclusively resides in the left arm. Further, the 5’-PPP is required for activity. Finally, we identified Alu RNAs with altered activity. Changing nucleotide sequence *via* synthetic methods produced Alu RNAs with increased activity. We also identified native Alu RNAs that stimulated IRF transcriptional activity but failed to stimulate NF-kB activity.

Alu RNAs are about 300 nucleotides in length. Our analysis of A-to-I editing of Alu RNAs shows that only about 5-8 A’s are edited to I’s. For the most part, edits are located in the same general region, towards the 3’ end of the left arm. Testing of synthetic unedited and edited Alu RNAs demonstrates that these low levels of editing are sufficient to largely abrogate activity. This level of editing also produces marked changes in the predicted structures. Although it is uncertain if it is possible to accurately predict changes in function from these predicted changes in structure, it does appear that the unedited Alu left arm possesses a more continuous double-stranded structure while the edited left arm seems divided into three distinct short double-stranded structures. It does seem noteworthy that changing just 5 A’s to G’s produces such a marked change in predicted structure.

Changes in levels of endogenous Alu dsRNAs may have significant human health consequences. For example, increased levels of Alu dsRNAs are seen in inflammatory diseases, MS (12), IBD (this study), SLE (49) in response to severe viral disease (10), and in certain forms of macular degeneration (31), and may be major drivers of the IRF and NF-kB driven transcriptional responses that are observed in these diseases and are thought to contribute to pathogenesis. Our results show that edited Alu dsRNAs inhibit transcriptional responses stimulated by unedited Alu dsRNAs suggesting that edited dsRNAs may also interact with dsRNA sensors and limit their ability to be activated by unedited Alu dsRNAs. It seems reasonable to propose that this might represent a therapeutic target to reduce Alu dsRNA-driven transcriptional responses and inflammation in those diseases where this response is potentially pathogenic.

Our studies also demonstrate that activity of Alu dsRNAs can be increased by altering nucleotide sequence. In addition, in our studies we identified two distinct functional classes of Alu dsRNAs; one class activates both IRF and NF-kB driven transcriptional responses, an example referred to in the text as AluJb, while the second class only activates IRF driven transcriptional responses, an example is Alu5. In general terms, dsRNA sensors, RIG-I, TLR3, and MDA5 activate both IRF and NF-kB transcriptional responses (19). One possible explanation seems to be that additional dsRNA sensors may exist that only activate IRF driven transcriptional responses and these sensors are preferentially activated by Alu5 while AluJb may preferentially activate dsRNA sensors that stimulate both ISRE and NF-kB driven transcriptional responses. Thus, AluJb may activate one or more dsRNA sensors resulting in activation of both IRF and NF-kB driven transcriptional responses while Alu5 may activate one or more sensors that results in selective activation of ISRE driven transcriptional responses. Thus, the sum of the total IRF and NF-kB transcriptional responses driven by Alu RNAs may be explained by existence of multiple dsRNA sensors, some that activate both transcriptional responses and some that activate only IRF or only NF-kB driven transcriptional responses.

It seems also that identification of Alu RNAs that selectively activate ISRE and NF-kB transcriptional responses versus those that activate only ISRE responses may have additional implications. In general terms, TLR agonists are attractive candidates as vaccine adjuvants by virtue of their ability to stimulate innate immune responses and therefore shape adaptive immune responses (50–53). However, excessive inflammation induced by TLR agonists has limited their development (54–56). In fact, a recent study shows that modulation of NF-kB responses induced by TLR agonists may improve vaccine tolerability and increase protection (57, 58). As such, Alu RNAs that selectively activate IRF transcriptional paths in the absence of activation of NF-kB transcriptional paths may provide vaccine adjuvant activity with reduced side effects. A similar case may be made for use of Alu RNAs that selectively activate IRF transcriptional paths in the absence of NF-kB activation as cancer immunotherapies, either alone or to augment existing immune-based therapies. Similar arguments may be made for an alternative to the use of type 1 IFNs to modulate viral infections or autoimmune disorders.

## Data Availability Statement

The datasets presented in this study can be found in online repositories. The names of the repository/repositories and accession number(s) can be found in the article/**Supplementary Material**.

## Ethics Statement

The studies involving human participants were reviewed and approved by Vanderbilt University Medical Center Institutional Review Board. The patients/participants provided their written informed consent to participate in this study.

## Author Contributions

Conceptualization, TA. Methodology, TA, JT, and PC. Formal analysis, TA, JT, RH, and KP. Investigation, JT, PC, RH, and KP. Writing: original draft, TA. Writing: review and editing, TA, JT, RH, KP, and PC. Supervision, TA and JT. Project administration, TA. Funding acquisition, TA. All authors contributed to the article and approved the submitted version.

## Funding

Supported by grants from the National Institutes of Health to TMA (R01AI044924, R21AI144193) and by a grant from the Pfizer Aspire program to TA. The funder was not involved in the study design, collection, analysis, interpretation of data, the writing of this article or the decision to submit it for publication.

## Conflict of Interest

The authors declare that the research was conducted in the absence of any commercial or financial relationships that could be construed as a potential conflict of interest.

## Publisher’s Note

All claims expressed in this article are solely those of the authors and do not necessarily represent those of their affiliated organizations, or those of the publisher, the editors and the reviewers. Any product that may be evaluated in this article, or claim that may be made by its manufacturer, is not guaranteed or endorsed by the publisher.

## Abbreviations

SINE, short interspersed nuclear element; dsRNA, double-stranded RNA; UTR, untranslated region; ISGs, interferon stimulated genes; HC, healthy control; COV-S, severe COVID-19 disease; COV-M, mild COVID-19 disease; FLU-S, severe influenza; MS, multiple sclerosis; IBD, inflammatory bowel disease; UC, ulcerative colitis; Cr, Crohn’s disease; Ce, Celiac disease; IBS, irritable bowel syndrome; DC, dendritic cells; NHBE, normal human lung epithelial cells; FDR, false discovery rate; ISRE, interferon stimulated response element.
