# Supplementary figures and images for: Alu RNA Structural Features Modulate Immune Cell Activation and A-to-I Editing of Alu RNAs Is Diminished in Human Inflammatory Bowel Disease
Source: Front Immunol. 2022 Jan 20;13:818023. doi: 10.3389/fimmu.2022.818023 (PMC8813004; doi:10.3389/fimmu.2022.818023)

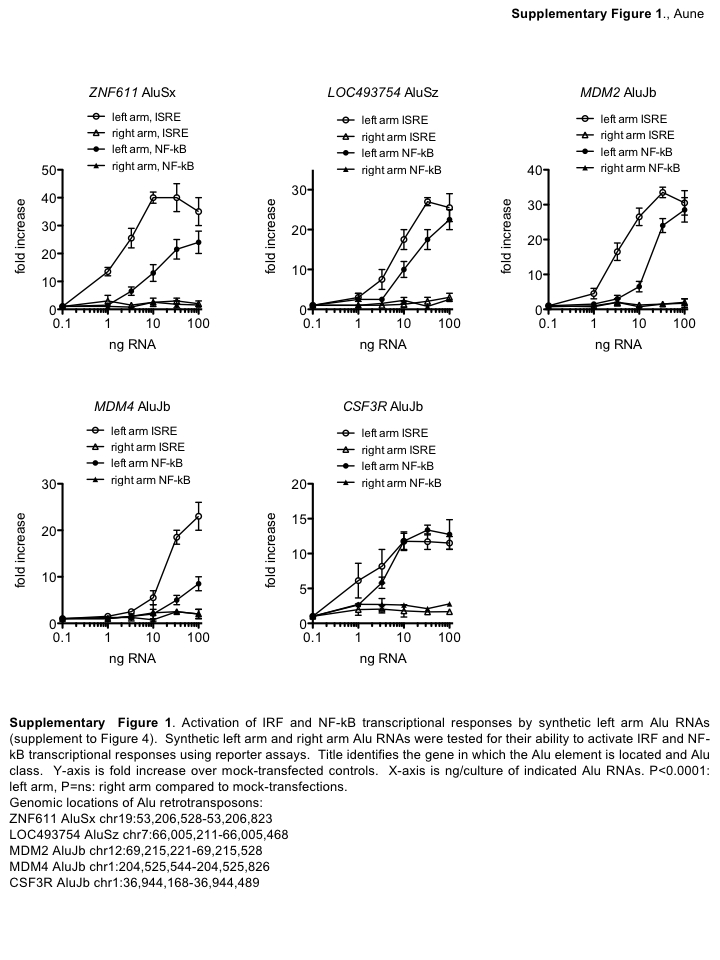

Supplement: Supplementary file 1 [file Image_1.tiff]
